# Lateralized position of femoral and tibial components during posterior-stabilized total knee arthroplasty leads to better functional outcomes

**DOI:** 10.1186/s43019-025-00275-4

**Published:** 2025-05-20

**Authors:** Shinichiro Nakamura, Yoshihisa Tanaka, Shinichi Kuriyama, Kohei Nishitani, Yugo 侑吾 Morita, Yugo 悠吾 Morita, Sayako Sakai, Yuki Shinya, Shuichi Matsuda

**Affiliations:** Department of Orthopaedic Surgery, Graduate School of Medicine, Kyoto University, 54 Shogoin-Kawaharacho, Sakyo-Ku, Kyoto, 606-8507 Japan

**Keywords:** Total knee arthroplasty, Mediolateral position, Functional outcomes, Muscle strength

## Abstract

**Background:**

The mediolateral position and postoperative translation of the femoral and tibial components relative to the respective bones after total knee arthroplasty (TKA) have not yet been investigated. The purpose of the current study was to investigate the effect of the mediolateral position of the femoral and tibial components on clinical outcomes including muscle strength and ambulatory function.

**Methods:**

A total of 86 consecutive knees were included. The mediolateral positions of the femoral and tibial components were measured on the postoperative long-leg radiographs. The mediolateral position of the femoral and tibial components was defined relative to the femoral distal anatomical axis and the tibial mechanical axis. The lateral position of the component was denoted as positive. The lateral translation of the femoral and tibial components was defined as the distance between the preoperative femoral and tibial centers and the postoperative center of the respective component. The Knee Society Score (KSS), New Knee Society Score (2011 KSS), and the Timed Up and Go (TUG) test results were evaluated 2 years postoperatively. Spearman’s correlation coefficient was calculated.

**Results:**

The lateral position of the femoral component was significantly positively correlated with KSS function score (*ρ* = 0.250, *p* = 0.020), 2011 KSS functional activities (*ρ* = 0.258, *p* = 0.017), and TUG values (*ρ* = − 0.241, *p* = 0.027). The lateral translation of the tibial component was significantly correlated with knee extension strength (*ρ* = 0.259, *p* = 0.017).

**Conclusions:**

The lateralized position of the femoral and tibial components positively influenced postoperative knee function. When the width of the component does not fit the resected surface, a lateralized position of the femoral and tibial components with respect to the respective bones can be recommended for better functional outcomes.

## Introduction

In total knee arthroplasty (TKA), the angular rotation and position of the femoral and tibial components are defined in three dimensions (3D). Angular rotation of implants along the three axes has been widely investigated [1–11]. The position in the proximal–distal axis alters the joint line and joint gap, and this affects the range of motion of the knee, postoperative pain, and premature component wear [12–15]. The position of the femoral component in the anterior–posterior axis alters the posterior condylar offset and anterior condylar height, thus affecting the flexion gap and patellofemoral contact force, respectively [16–18].

The position on the mediolateral axis can affect the relative positions of the femoral and tibial components and the condition of the surrounding soft tissues and muscles. In the femoral component, the position can be modified when the size of the femoral component is smaller than that of the resected surface in the mediolateral direction. In the tibial component, lateralization of the tibial baseplate and resection of the uncovered medial plateau are frequently carried out for severe varus deformities to achieve deformity correction and soft tissue balance of the medial gap [19–21].

The effect of the mediolateral position has been investigated in biomechanical studies using a weight-bearing knee rig [22, 23]. Compared with medialization of the femoral component, there was a slight decrease in retropatellar peak pressure (medial 6.5 MPa versus lateral 6.0 MPa) and a greater rollback (medial 7.2 mm versus lateral 8.8 mm) after lateralization of the femoral component [23]. Compared with medialization of the tibial component, there was a slight decrease in the retropatellar peak pressure (medial 7.5 MPa versus lateral 7.2 MPa) and reduced external rotation of the femoral component (medial 7.0° versus lateral 3.9°) in the lateralized tibial component version [22]. However, the effect of the mediolateral position on clinical outcomes remains unknown.

The purpose of the current study was to investigate the effect of the mediolateral position of the femoral and tibial components on clinical outcomes, including muscle strength and ambulatory function. We hypothesized that the mediolateral position of the implant could affect clinical outcomes.

## Materials and methods

**Fig. 1:**
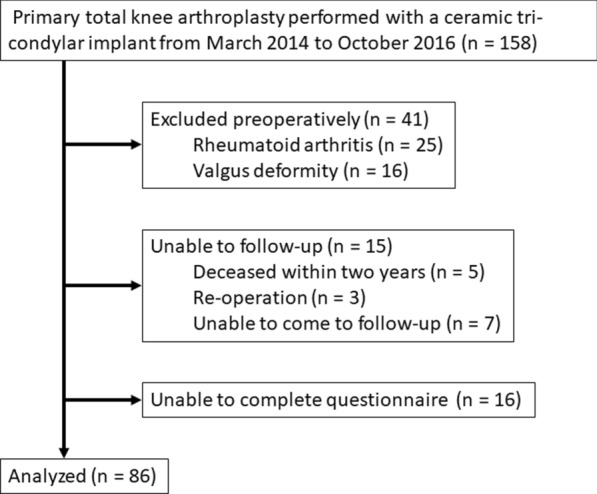
Flow chart of the study

The current study was conducted using consecutive primary TKA interventions carried out with a ceramic tricondylar implant (Bi-Surface 5; Kyocera, Kyoto, Japan) from March 2014 to October 2016; data were collected prospectively and retrospectively analyzed. The sole inclusion criterion was primary TKA for medial knee osteoarthritis. Patients with valgus deformity or a confirmed diagnosis of secondary inflammatory knee arthritis were excluded from the study. Patients were also excluded if they had undergone revision TKA or if they had not provided patient-reported outcomes or had been absent from muscle strength assessments (Fig. 1). Knees from a total of nine men and 77 women with an average age of 76.1 years (standard deviation [SD] = 6.9 years) were included in the study. The mean preoperative hip–knee–ankle (HKA) angle, based on the angle between the mechanical axis of the femur and tibia, was 12.4° varus (SD = 4.5°). The mean height and weight were 150.8 cm (SD = 6.8 cm) and 61.0 kg (SD = 11.2 kg), respectively, and the mean body mass index was 26.7 kg/m^2^ (SD = 4.1). Informed consent was obtained from all patients. The study design was approved by the institutional ethics review board and all procedures were performed in accordance with the ethical standards of the institutional research committee.

TKA was performed using the mechanical alignment method in a uniform manner with the goal of neutral alignment in the coronal plane. The ceramic tricondylar implant we used has a ball-and-socket joint as a third condyle in the midposterior portion to induce femoral rollback [24, 25]. In previous biomechanical studies, the tricondylar implant showed similar posterior translation and similar axial rotation in comparison with other posterior-stabilized implants, and the ball-and-socket joint has been proven to function as a postcam mechanism in vivo [26–29]. The medial parapatellar approach was used, and the posterior cruciate ligament (PCL) was sacrificed. Bone resection was conducted using the following measured resection technique. The distal femur was cut perpendicular to its mechanical axis. Regarding the rotational alignment of the femoral component, the angle between the surgical epicondylar axis, defined as the line connecting the lateral epicondylar prominence and the medial sulcus of the medial epicondyle and the posterior condylar axis, was measured preoperatively using computed tomography, and this measurement was employed while using the cutting jig relative to the posterior condylar axis. The proximal tibia was cut perpendicular to the mechanical axis in the coronal plane. In the sagittal plane, the posterior slope was set at 5° relative to the tibial shaft. The rotational alignment of the tibial component was determined with reference to the anteroposterior axis of the tibia (the Akagi’s line) [30]. The mediolateral position of the femoral component was determined on the basis of the intercondylar notch and the resected bony surface so that the intercondylar notch was centered on the femoral component while avoiding the overhang of the femoral component on the anterior flange. Tibial osteophytes were first resected thoroughly, and then the appropriate implant size was positioned to fit the lateral end of the tibial bone and the tibial component. If the medial tibia bone was more elevated than the tibial component, further medial bone was resected to the tibial component. The tibial bone beyond the component on the medial side, was finally resected. With regard to ligament balancing, the deep layer of the medial collateral ligament was released within 1 cm of the joint line for bone and osteophyte resection. Extensive medial release was not performed, even if lateral laxity was observed during extension and/or flexion. A patellar resurfacing was conducted in all knees. All components were fixed with Simplex bone cement (Stryker, Kalamazoo, MI, USA).

**Fig. 2:**
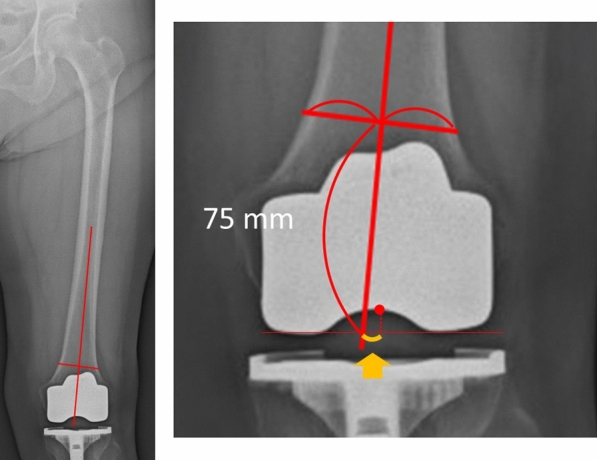
The medial–lateral position of the femoral component was defined as the distance between the distal femoral axis (solid line) and the center of the femoral component (dotted line) based on the tangent line of the femoral component

**Fig. 3:**
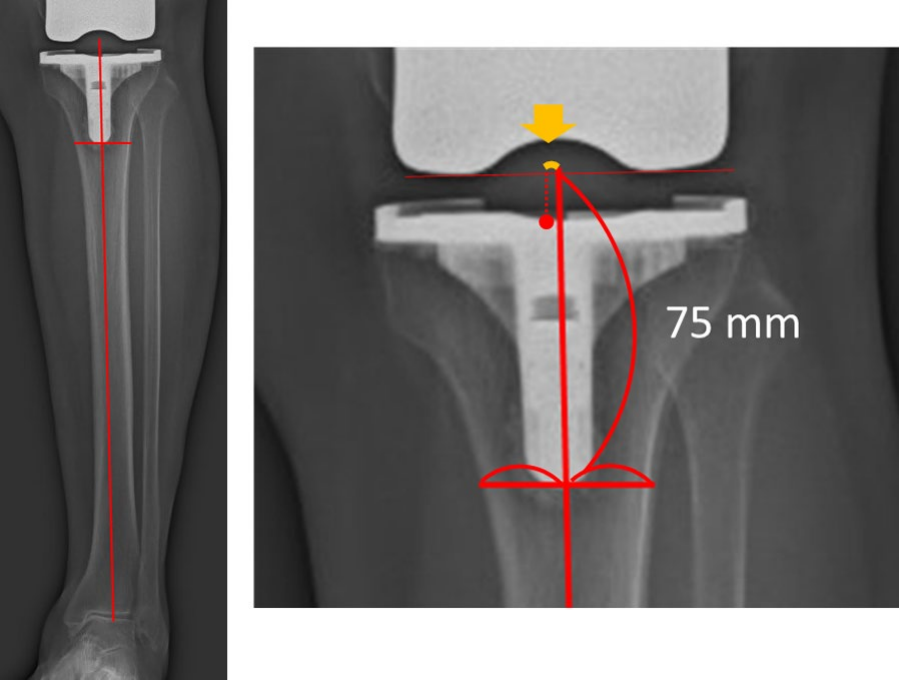
The medial–lateral position of the tibial component was defined as the distance between the tibial axis (solid line) and the center of the tibial component (dotted line) based on the tangent line of the femoral component

The mediolateral positions of the femoral and tibial components were measured post-TKA on standing anteroposterior long-leg radiographs. During radiography, the patients stood with the patellae facing forward, feet straight, and shoulders wide. The distal femoral axis was defined as the line passing the midpoint of the center of the femoral shaft and the midpoint of the femoral shaft 7.5 cm proximal to the joint line [31, 32]. The center of the femoral component was defined as the most concave position. The mediolateral position of the femoral component was defined as the distance between the distal femoral axis and the center of the femoral component along the tangent line of the femoral component (Fig. 2). The lateral position of the femoral component relative to the distal femoral axis was denoted as positive. The tibial axis was defined as the line passing through the midmalleolar point and the midpoint of the tibial shaft 7.5 cm distal to the joint line [31, 32]. The center of the tibial component was defined as the center of the tibial tray (Fig. 3). The mediolateral position of the tibial component was defined as the distance between the tibial axis and the center of the tibial component along the tangent line of the femoral component. The lateral position of the tibial component relative to the tibial axis was denoted as positive. Femoral and tibial implant overhanging in the medial–lateral direction was measured at the bone resection level.

**Fig. 4:**
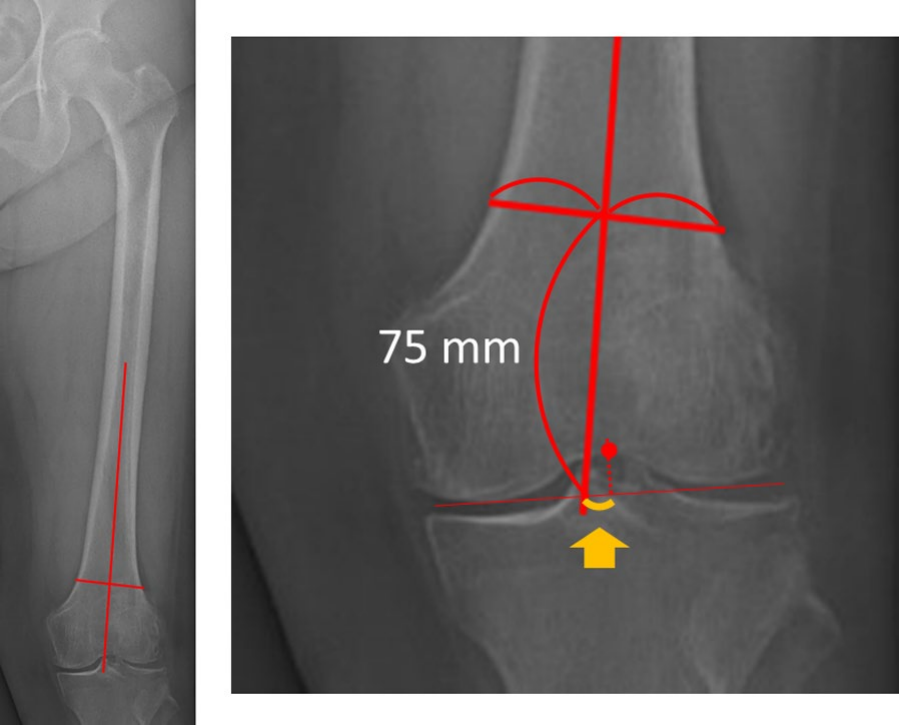
The medial–lateral position of the femoral center was defined as the distance between the distal femoral axis (solid line) and the center of the femur (dotted line) based on the tangent line of the femur

**Fig. 5:**
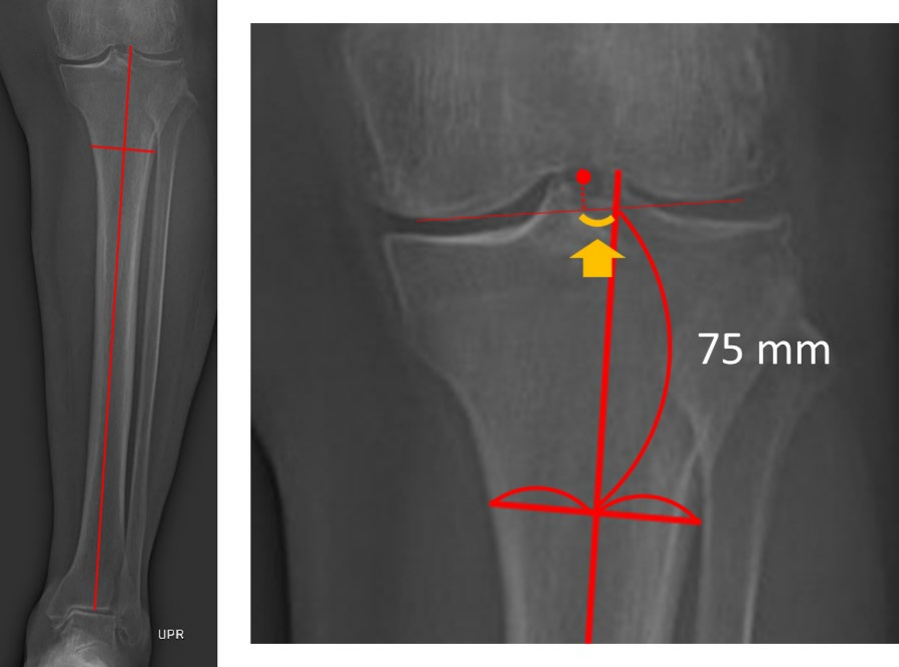
The medial–lateral position of the tibial center was defined as the distance between the tibial axis (solid line) and the center of the tibia (dotted line) based on the tangent line of the femur

In the preoperative radiograph, the mediolateral positions of the femoral and tibial centers relative to the distal femoral and tibial axes, respectively, were measured in the same manner (Figs. 4 and 5). The translation of the femoral and tibial components relative to the respective bone centers was calculated. Lateral translation of the femoral and tibial components was denoted as positive.

The same postoperative rehabilitation program was applied to all patients. Physical therapists continued knee flexion exercises and muscle strength training for 1 month. Following the completion of the rehabilitation program, patients were free to perform deep flexion activities and were able to perform most activities without any restrictions, including deep knee flexion. Physical examinations and knee scoring were conducted 2 years postoperatively using the Knee Society Score (KSS) [33] and the New Knee Society Score (2011 KSS) [34, 35]. The range of motion of the knee was passively measured with a long-arm goniometer in the supine position. Postoperative examinations were performed by physical therapists, independent of the surgeons performing the surgeries. For muscle strength evaluation, knee extension and flexion muscle strengths were measured using the IsoForce GT-330 instrument (OG Giken Co. Ltd., Okayama, Japan) in the open kinetic chain. A force sensor was placed on the lower leg 5 cm above the malleolus, with the patient in a sitting position. Torque was calculated by multiplying the force by the lever arm (distance between the position of the force sensor and the lateral epicondyle of the femur) and was expressed as a percentage of body weight (Nm/kg) [36–38]. Muscle strength was measured twice, and the highest values were recorded. For ambulatory function, the 10 m walking time (10 m WT) and the Timed Up and Go (TUG) tests were conducted. Each patient walked as fast as possible without using appropriate assistive devices such as a cane, crutches, or a walker. In the 10 m WT test, the time taken to walk 10 m away from the standing position was measured. In the TUG test, the time required to perform the following series of actions was measured: standing up from a chair, walking 3 m, changing direction, walking back to the chair, and sitting down.

### Data analyses

All analyses were conducted using JMP Pro software, version 17.0.0 (SAS Institute Inc., Cary, NC, USA). To test the interobserver intraclass correlation coefficients (ICC) for the mediolateral positions of the femoral and tibial components, all measurements were repeated in ten randomly selected knees by another orthopaedic surgeon (as a second observer). To test intraobserver reliability, measurements were repeated 4 weeks apart. Spearman’s rank correlation coefficients were calculated to determine the correlations between the position and translation of the femoral and tibial components and the KSS and 2011 KSS subscales, muscle strength, and ambulatory function. Comparisons between knees with and without overhanging were made using Mann–Whitney *U* test. The level of significance was set at *p* < 0.05. The sample size required to detect a weak correlation (*r* = 0.3) (two-sided, *α* = 0.05, power = 80%) was estimated to be 82 knees using G*power 3.1.9.7 software (Heinrich-Heine-Universität Düsseldorf, Düsseldorf, Germany).

## Results

The mean mediolateral position of the femoral component was 1.9 mm (SD = 1.8 mm) lateral relative to the distal femoral axis. The mean mediolateral position of the tibial component was 0.3 mm (SD = 2.4 mm) lateral relative to the tibial axis. The inter- and intra-observer reliabilities (ICC values) of each variable were robust (interobserver reliability: 0.941 and 0.957 for the femoral and tibial components, respectively; intraobserver reliability: 0.967 and 0.960 for the femoral and tibial components, respectively). The center of the femoral component was translated 1.9 mm (SD = 1.9 mm) medial relative to the preoperative femoral center. The center of the tibial component was translated 3.0 mm (SD = 3.0 mm) lateral relative to the preoperative tibial center. Medial tibial overhanging of more than 3 mm was seen in four knees, while lateral tibial overhanging was seen in three knees. Femoral overhanging was not seen.

The mean values for the clinical outcomes, including the KSS, 2011 KSS, muscle strength, and ambulatory function values showed robust results (Table 1). The mean knee and functional KSS scores were 91.7 (SD = 10.4) and 78.7 (SD = 20.2), respectively.

**Table 1:** Postoperative clinical outcomes at 2 years

	Average	SD
KSS knee score	91.7	10.4
KSS function score	78.7	20.2
2011 KSS symptoms	21.3	4.3
2011 KSS satisfaction	27.1	7.3
2011 KSS expectation	9.9	2.7
2011 KSS functional activities	64.6	19.5
Knee extension strength (Nm/kg)	1.16	0.48
Knee flexion strength (Nm/kg)	0.45	0.25
Timed up and go test (s)	9.6	3.8
10 m walking time (s)	8.6	3.4
Knee extension angle (degrees)	−2.6	4.8
Knee flexion angle (degrees)	119.3	12.7

*KSS* Knee Society Score, *SD* standard deviation

**Fig. 6:**
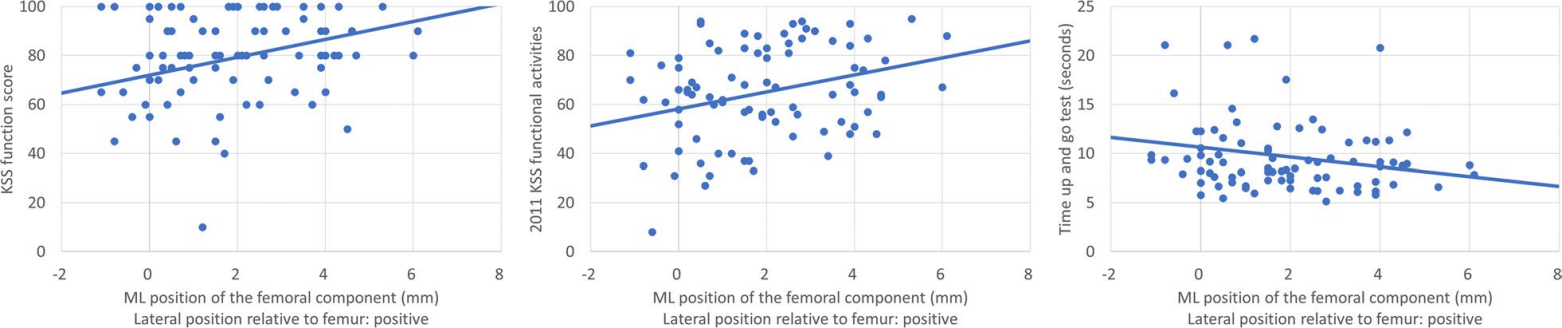
Correlation between the medial–lateral position of the femoral component and Knee Society Score function score, 2011 Knee Society Score functional activities, and the timed up and go test

The mediolateral position of the femoral component was significantly correlated with KSS function score (*ρ* = 0.250, *p* = 0.020), 2011 KSS functional activities (*ρ* = 0.258, *p* = 0.017), and TUG values (*ρ* = −0.241, *p* = 0.027) (Fig. 6). The lateral position of the femoral component positively affected knee function. The mediolateral position of the tibial component did not correlate with clinical outcomes (Table 2).

**Table 2:** Correlation between medial–lateral position of the femoral and tibial components and clinical outcomes

	Femoral component		Tibial component	
	Coefficient	*p*-Value	Coefficient	*p*-Value
KSS knee score	0.002	0.987	0.078	0.476
KSS function score	***0.250***	***0.020***	−0.046	0.673
2011 KSS symptoms	0.089	0.414	0.161	0.140
2011 KSS satisfaction	0.129	0.238	0.134	0.219
2011 KSS expectation	0.043	0.695	0.071	0.518
2011 KSS functional activities	***0.258***	***0.017***	0.050	0.648
Knee extension strength (Nm/kg)	0.123	0.263	−0.024	0.830
Knee flexion strength (Nm/kg)	0.180	0.099	−0.022	0.843
Time up and go test (s)	−***0.241***	***0.027***	0.129	0.238
10 m walking time (s)	−0.176	0.108	0.134	0.223
Knee extension angle (degrees)	−0.067	0.550	−0.020	0.859
Knee flexion angle (degrees)	−0.011	0.924	−0.099	0.372

Bold italic values indicated statistical significance

*KSS* Knee Society Score

**Fig. 7:**
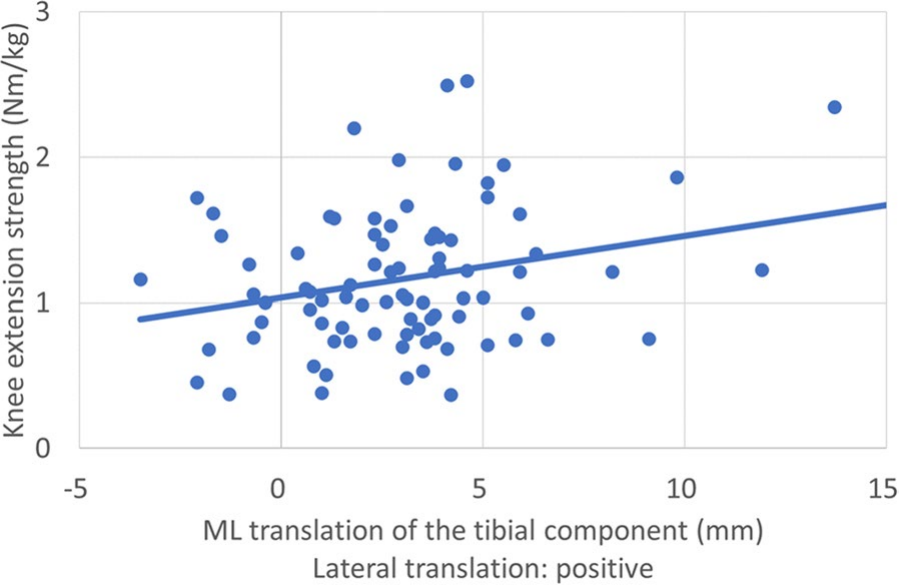
Correlation between the medial–lateral translation of the tibial component and knee extension strength

The mediolateral translation of the femoral component did not correlate with clinical outcomes, muscle strength, or ambulatory function. The mediolateral translation of the tibial component was significantly correlated with knee extension strength (*ρ* = 0.259, *p* = 0.017) (Table 3, Fig. 7). The knees with tibial overhanging had similar clinical outcomes, muscle strength, and ambulatory function compared with the knees without tibial overhanging.

**Table 3:** Correlation between medial–lateral translation of the femoral and tibial components and clinical outcomes

	Femoral component		Tibial component	
	Coefficient	*p*-Value	Coefficient	*p*-Value
KSS knee score	−0.131	0.228	0.027	0.806
KSS function score	0.097	0.376	0.048	0.663
2011 KSS symptoms	0.020	0.858	0.050	0.648
2011 KSS satisfaction	0.067	0.538	−0.019	0.861
2011 KSS expectation	0.032	0.771	0.036	0.743
2011 KSS functional activities	0.077	0.483	0.119	0.275
Knee extension strength (Nm/kg)	−0.061	0.579	***0.259***	***0.017***
Knee flexion strength (Nm/kg)	−0.021	0.848	0.142	0.195
Time up and go test (s)	−0.121	0.272	−0.130	0.236
10 m walking time (s)	−0.093	0.397	−0.077	0.482
Knee extension angle (degrees)	−0.022	0.842	−0.086	0.439
Knee flexion angle (degrees)	−0.269	0.014	0.041	0.714

Bold italic values indicated statistical significance

*KSS* Knee Society Score

## Discussion

The mediolateral position of the femoral and tibial components has not been fully investigated, and its effect on clinical outcomes remains unknown. In the current study, the mediolateral position was measured after TKA, and the correlation between the position and clinical outcomes was evaluated. The lateral position of the femoral component positively influenced the KSS function score, 2011 KSS functional activities, and TUG values, while the lateral translation of the tibial component positively influenced knee extension strength. The hypothesis that the mediolateral position of the implant can affect clinical outcomes was confirmed. When the width of the component is smaller than that of the resected surface, aligning the lateral edges of the femoral and tibial components to the respective bones can be recommended for better functional outcomes.

The angular rotation and position of the femoral and tibial components relative to the respective bones were defined in 3D. The angular rotation of implants has been commonly investigated in the coronal, axial, and sagittal planes. Conventionally, attention has been paid to rotation in the coronal plane because of equal load distribution in the medial and lateral compartments and subsequent long-term durability. Axial plane rotation affects patellar tracking after TKA. The proximal–distal position of the implants affects the joint line and joint gap. The anterior–posterior position of the implant, especially the femoral component, is related to the flexion gap that can affect postoperative knee flexion. However, the mediolateral position that can affect clinical outcomes after TKA has not been investigated.

Defining the mediolateral position of the implant after TKA is challenging. In addition, preoperative deformities and bone defects can prevent the comparison of the mediolateral position of the knee joint between the pre- and post-operative conditions. In the current study, the mediolateral position of the femoral and tibial components was defined relative to the femoral distal anatomical axis and the tibial mechanical axis, respectively. The translation of the femoral and tibial components relative to the respective centers was calculated on the basis of the preoperative femoral and tibial centers. The effect of osteophyte resection during TKA can be eliminated by referring to the femoral distal anatomical axis and the tibial mechanical axis. Considering individual bone morphology, femoral lateral bowing and steeping of the tibial plateau inclination might affect the medial–lateral position and translations [31]. This is the reason why a number of cases showed negative (medial) positions, although we aimed to the lateralized position.

The mediolateral position of the femoral component can alter patellar tracking. A study using fresh cadaver specimens demonstrated that, following mediolateral translation of the femoral component, the patella significantly shifted and tilted in the same direction [22]. In a simulation of knee radiographs of young patients, the estimated mean lateral shift for all cases was 5.99 mm, suggesting that the coronal positional relationship between the femur and tibia is altered, and subsequent ligament imbalance may occur [39]. The lateralized position of the femoral component can prevent a lateral shift of the femur that enables preservation of the surrounding ligament tension and patellar tracking. In the current study, the lateral position of the femoral component tended to increase knee muscle strength that can lead to better KSS function scores, 2011 KSS functional activities, and TUG test results.

The mediolateral position of the tibial component can also affect patellofemoral kinematics. The medial position of the tibial component causes medialization of the femoral component, and this leads to an increase in the tibial tuberosity–trochlear groove (TT–TG) distance and subsequent patellar lateral tilt and shift [40]. In the current study, lateral translation of the tibial component correlated positively with knee extension strength (*ρ* = 0.259, *p* = 0.017), which might be due to adequate patellar tracking.

The current study has several limitations. This study used a single ceramic posterior-stabilized implant design with limited size variation, and the results cannot be applied to other implants such as cruciate-retaining implants, modern implants with large size variations, or cobalt-chrome implants. Additionally, most of the knees included in the current study were of women because consecutive TKA patients were recruited without any adjustment for sex. In addition, the effects of component overhangs were only investigated at the bone resection level in the medial–lateral direction. An overhang of the femoral and tibial components leads to poor clinical outcomes with more pain [41, 42]. Our findings suggested a lateralized position rather than an overhang of the femoral and tibial components. We did not investigate other angular rotations, proximal–distal, or anterior–posterior positions of the femoral and tibial components relative to the respective bone and rotations between the femoral and tibial components owing to lack of postoperative computed tomography. The mediolateral positioning of the femur and tibia can be influenced by rotational alignment. Additionally, the preoperative and postoperative positions on standing radiographs can be altered, affecting femoral bowing.

## Conclusions

The lateralized positions of the femoral and tibial components positively influenced postoperative knee function. When the width of the component does not fit the resected surface, aligning the lateral edges of the femoral and tibial components to the respective bones can be recommended for better functional outcomes.

## Data Availability

My manuscript has no associated data.
